# 5-Chloro-2-(2-fluoro­phen­yl)-7-methyl-3-methyl­sulfinyl-1-benzo­furan

**DOI:** 10.1107/S1600536814011635

**Published:** 2014-05-24

**Authors:** Hong Dae Choi, Uk Lee

**Affiliations:** a Department of Chemistry, Dongeui University, San 24 Kaya-dong, Busanjin-gu, Busan 614-714, Republic of Korea; b Department of Chemistry, Pukyong National University, 599-1 Daeyeon 3-dong, Nam-gu, Busan 608-737, Republic of Korea

## Abstract

In the title compound, C_16_H_12_ClFO_2_S, the dihedral angle between the mean planes of the benzo­furan and 2-fluoro­phenyl rings is 34.85 (6)°. In the crystal, mol­ecules are linked *via* pairs of C—H⋯O hydrogen bonds, forming zigzag chains along [001]. The chains are linked by C—H⋯π inter­actions, forming a three-dimensional structure.

## Related literature   

For background information and the crystal structures of related compounds, see: Choi *et al.* (2010 ▶, 2013 ▶).
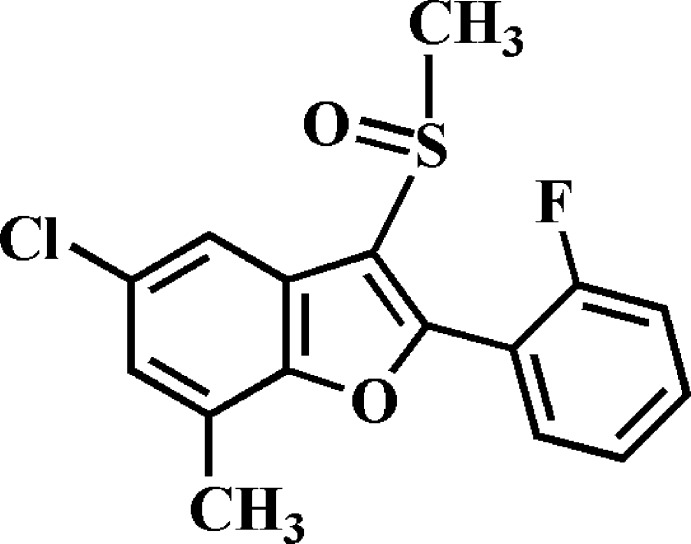


## Experimental   

### 

#### Crystal data  

C_16_H_12_ClFO_2_S
*M* *_r_* = 322.77
Monoclinic, 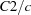

*a* = 11.3980 (2) Å
*b* = 15.7819 (4) Å
*c* = 16.7231 (4) Å
β = 104.370 (1)°
*V* = 2914.07 (11) Å^3^
*Z* = 8
Mo *K*α radiation
μ = 0.42 mm^−1^
*T* = 173 K
0.55 × 0.35 × 0.33 mm

#### Data collection  

Bruker SMART APEXII CCD diffractometer
Absorption correction: multi-scan (*SADABS*; Bruker, 2009 ▶) *T*_min_ = 0.678, *T*_max_ = 0.746
13530 measured reflections
3601 independent reflections
3105 reflections with *I* > 2σ(*I*)
*R*_int_ = 0.024

#### Refinement  

*R*[*F* ^2^ > 2σ(*F* ^2^)] = 0.037
*wR*(*F* ^2^) = 0.098
*S* = 1.04
3601 reflections
192 parameters
H-atom parameters constrained
Δρ_max_ = 0.43 e Å^−3^
Δρ_min_ = −0.37 e Å^−3^

### 

Data collection: *APEX2* (Bruker, 2009 ▶); cell refinement: *SAINT* (Bruker, 2009 ▶); data reduction: *SAINT*; program(s) used to solve structure: *SHELXS97* (Sheldrick, 2008 ▶); program(s) used to refine structure: *SHELXL97* (Sheldrick, 2008 ▶); molecular graphics: *ORTEP-3 for Windows* (Farrugia, 2012 ▶) and *DIAMOND* (Brandenburg, 1998 ▶); software used to prepare material for publication: *SHELXL97*.

## Supplementary Material

Crystal structure: contains datablock(s) I. DOI: 10.1107/S1600536814011635/cv5456sup1.cif

Structure factors: contains datablock(s) I. DOI: 10.1107/S1600536814011635/cv5456Isup2.hkl

Supporting information file. DOI: 10.1107/S1600536814011635/cv5456Isup3.cml

CCDC reference: 1004137

Additional supporting information:  crystallographic information; 3D view; checkCIF report

## Figures and Tables

**Table 1:** Hydrogen-bond geometry (Å, °) *Cg*1 is the centroid of the C1/C2/C7/O1/C8 furan ring.

*D*—H⋯*A*	*D*—H	H⋯*A*	*D*⋯*A*	*D*—H⋯*A*
C5—H5⋯O2^i^	0.95	2.44	3.2916 (19)	149
C15—H15*A*⋯O2^i^	0.98	2.45	3.347 (2)	152
C16—H16*B*⋯*Cg*1^ii^	0.98	2.92	3.641 (2)	131

Symmetry codes: (i) 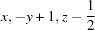; (ii) 
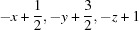
.

**Fig. 1.**
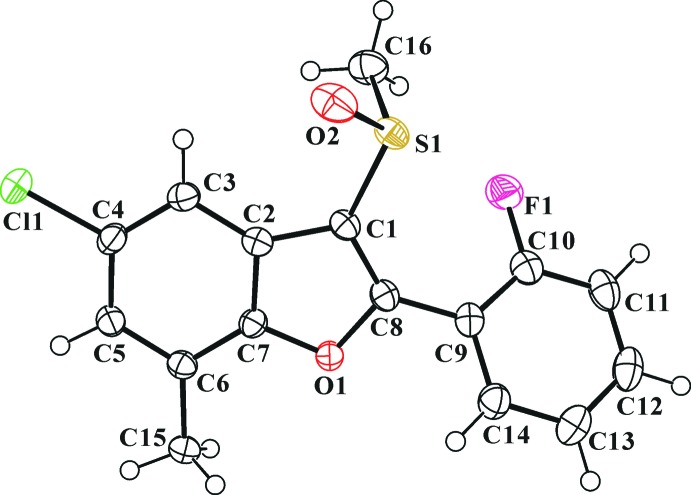
The molecular structure of the title compound showing the atom numbering scheme. The displacement ellipsoids are drawn at the 50% probability level. The hydrogen atoms are presented as small spheres of arbitrary radius.

**Fig. 2.**
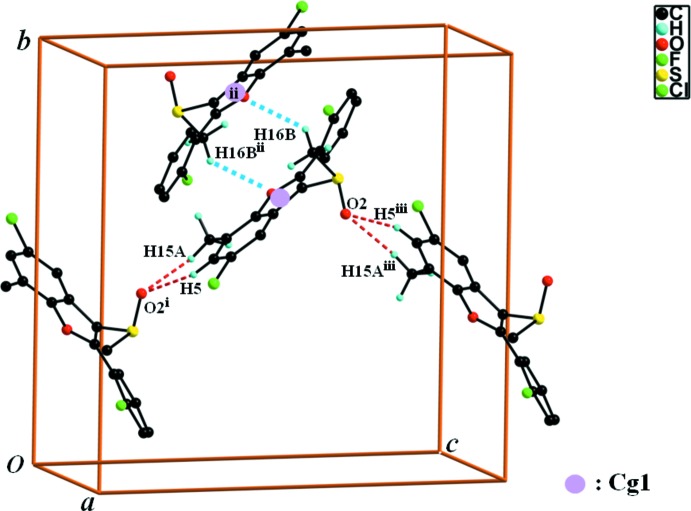
A view of the C—H···O and C—H···π interactions (dotted lines) in the crystal structure of the title compound. H atoms not participating in hydrogen bonding were omitted for clarity [symmetry codes: (i) x, - y + 1, z - 1/2; (ii) - x + 1/2, - y + 3/2, - z + 1; (iii) x, - y + 1, z + 1/2].

## supplementary crystallographic information

### 1. Comment

As a part of our ongoing study of 5-chloro-7-methyl-3-methylsulfinyl-1-benzofuran derivatives containing 4-chlorophenyl (Choi *et al.*, 2010) and 4-fluorophenyl (Choi *et al.*, 2013) substituents in 2-position, we report here he crystal structure of the title compound.

In the title molecule (Fig. 1), the benzofuran unit is essentially planar, with a mean deviation of 0.004 (1) Å from the least-squares plane defined by the nine constituent atoms. The 2-fluorophenyl ring is essentially planar, with a mean deviation of 0.004 (1) Å from the least-squares plane defined by the six constituent atoms. The dihedral angle formed by the benzofuran ring system and the 2-fluorophenyl ring is 34.85 (6)°. In the crystal structure (Fig. 2), molecules are paired by C—H···π interactions (Table 1, Cg1 is the centroid of the C1/C2/C7/O1/C8 furan ring) into inversion dimers. These dimers are further linked by C—H···O hydrogen bonds (Table 1), forming a three-dimensional supramolecular network.

### 2. Experimental

3-Chloroperoxybenzoic acid (77%, 224 mg, 1.0 mmol) was added in small portions to a stirred solution of 5-chloro-2-(2-fluorophenyl)-7-methyl-3-methylsulfanyl-1-benzofuran (276 mg, 0.9 mmol) in dichloromethane (40 mL) at 273 K. After being stirred at room temperature for 6h, the mixture was washed with saturated sodium bicarbonate solution and the organic layer was separated, dried over magnesium sulfate, filtered and concentrated at reduced pressure. The residue was purified by column chromatography (hexane–ethyl acetate, 1:1 *v*/*v*) to afford the title compound as a colorless solid [yield 72%, m.p. 419–420 K; *R*_f_ = 0.51 (hexane-ethyl acetate, 1:1 *v*/*v*)]. Single crystals suitable for X-ray diffraction were prepared by slow evaporation of a solution of the title compound in ethyl acetate at room temperature.

### 3. Refinement

All H atoms were positioned geometrically and refined using a riding model, with C—H = 0.95 Å for aryl and 0.99 Å for methyl H atoms. *U*_iso_ (H) = 1.2*U*_eq_ (C) for aryl and 1.5*U*_eq_ (C) for methyl H atoms. The positions of methyl hydrogens were optimized using the SHELXL-97's command AFIX 137 (Sheldrick, 2008).

### Crystal data

**Table:**

C_16_H_12_ClFO_2_S	*F*(000) = 1328
*M**_r_* = 322.77	*D*_x_ = 1.471 Mg m^−^^3^
Monoclinic, *C*2/*c*	Melting point = 420–419 K
Hall symbol: -C 2yc	Mo *K*α radiation, λ = 0.71073 Å
*a* = 11.3980 (2) Å	Cell parameters from 6112 reflections
*b* = 15.7819 (4) Å	θ = 2.3–28.3°
*c* = 16.7231 (4) Å	µ = 0.42 mm^−^^1^
β = 104.370 (1)°	*T* = 173 K
*V* = 2914.07 (11) Å^3^	Block, colourless
*Z* = 8	0.55 × 0.35 × 0.33 mm

### Data collection

**Table:**

Bruker SMART APEXII CCD diffractometer	3601 independent reflections
Radiation source: rotating anode	3105 reflections with *I* > 2σ(*I*)
Graphite multilayer monochromator	*R*_int_ = 0.024
Detector resolution: 10.0 pixels mm^-1^	θ_max_ = 28.3°, θ_min_ = 2.3°
φ and ω scans	*h* = −15→15
Absorption correction: multi-scan (*SADABS*; Bruker, 2009)	*k* = −20→13
*T*_min_ = 0.678, *T*_max_ = 0.746	*l* = −22→22
13530 measured reflections	

### Refinement

**Table:**

Refinement on *F*^2^	Primary atom site location: structure-invariant direct methods
Least-squares matrix: full	Secondary atom site location: difference Fourier map
*R*[*F*^2^ > 2σ(*F*^2^)] = 0.037	Hydrogen site location: difference Fourier map
*wR*(*F*^2^) = 0.098	H-atom parameters constrained
*S* = 1.04	*w* = 1/[σ^2^(*F*_o_^2^) + (0.0488*P*)^2^ + 2.3447*P*] where *P* = (*F*_o_^2^ + 2*F*_c_^2^)/3
3601 reflections	(Δ/σ)_max_ = 0.001
192 parameters	Δρ_max_ = 0.43 e Å^−^^3^
0 restraints	Δρ_min_ = −0.37 e Å^−^^3^

### Special details

**Table:**

Geometry. All esds (except the esd in the dihedral angle between two l.s. planes) are estimated using the full covariance matrix. The cell esds are taken into account individually in the estimation of esds in distances, angles and torsion angles; correlations between esds in cell parameters are only used when they are defined by crystal symmetry. An approximate (isotropic) treatment of cell esds is used for estimating esds involving l.s. planes.
Refinement. Refinement of F^2^ against ALL reflections. The weighted R-factor wR and goodness of fit S are based on F^2^, conventional R-factors R are based on F, with F set to zero for negative F^2^. The threshold expression of F^2^ > 2sigma(F^2^) is used only for calculating R-factors(gt) etc. and is not relevant to the choice of reflections for refinement. R-factors based on F^2^ are statistically about twice as large as those based on F, and R- factors based on ALL data will be even larger.

### Fractional atomic coordinates and isotropic or equivalent isotropic displacement parameters (Å^2^)

**Table:**

	*x*	*y*	*z*	*U*_iso_*/*U*_eq_	
S1	0.32997 (4)	0.67149 (3)	0.68519 (2)	0.02744 (11)	
Cl1	0.08168 (4)	0.41662 (3)	0.42374 (3)	0.03932 (13)	
F1	0.41414 (9)	0.83843 (6)	0.64599 (7)	0.0411 (3)	
O1	0.48466 (9)	0.65185 (7)	0.49830 (6)	0.0241 (2)	
O2	0.29800 (12)	0.58583 (8)	0.71083 (7)	0.0400 (3)	
C1	0.37028 (13)	0.65573 (9)	0.59058 (8)	0.0234 (3)	
C2	0.31849 (13)	0.59295 (9)	0.52894 (8)	0.0229 (3)	
C3	0.22012 (13)	0.53726 (10)	0.51473 (9)	0.0257 (3)	
H3	0.1677	0.5342	0.5508	0.031*	
C4	0.20354 (13)	0.48715 (10)	0.44560 (9)	0.0265 (3)	
C5	0.27923 (13)	0.48869 (10)	0.39107 (9)	0.0258 (3)	
H5	0.2627	0.4524	0.3443	0.031*	
C6	0.37786 (13)	0.54272 (10)	0.40503 (8)	0.0235 (3)	
C7	0.39325 (12)	0.59329 (9)	0.47455 (9)	0.0223 (3)	
C8	0.46851 (13)	0.68894 (10)	0.56957 (8)	0.0239 (3)	
C9	0.55823 (13)	0.75389 (10)	0.60357 (9)	0.0254 (3)	
C10	0.52975 (15)	0.82705 (10)	0.64050 (9)	0.0301 (3)	
C11	0.61223 (17)	0.89018 (11)	0.67021 (10)	0.0364 (4)	
H11	0.5891	0.9393	0.6954	0.044*	
C12	0.72974 (17)	0.88051 (12)	0.66256 (11)	0.0392 (4)	
H12	0.7884	0.9231	0.6829	0.047*	
C13	0.76166 (16)	0.80876 (12)	0.62528 (11)	0.0397 (4)	
H13	0.8423	0.8025	0.6200	0.048*	
C14	0.67724 (14)	0.74612 (11)	0.59571 (10)	0.0324 (3)	
H14	0.7002	0.6974	0.5699	0.039*	
C15	0.46317 (14)	0.54748 (11)	0.34986 (9)	0.0279 (3)	
H15A	0.4437	0.5025	0.3083	0.042*	
H15B	0.5465	0.5403	0.3829	0.042*	
H15C	0.4550	0.6028	0.3223	0.042*	
C16	0.18856 (15)	0.72430 (12)	0.64558 (11)	0.0373 (4)	
H16A	0.1382	0.6906	0.6008	0.056*	
H16B	0.2033	0.7802	0.6244	0.056*	
H16C	0.1468	0.7310	0.6898	0.056*	

### Atomic displacement parameters (Å^2^)

**Table:**

	*U*^11^	*U*^22^	*U*^33^	*U*^12^	*U*^13^	*U*^23^
S1	0.0348 (2)	0.0279 (2)	0.02088 (18)	0.00321 (16)	0.00929 (14)	0.00125 (14)
Cl1	0.0300 (2)	0.0385 (2)	0.0518 (3)	−0.01287 (17)	0.01449 (17)	−0.01152 (19)
F1	0.0430 (6)	0.0310 (5)	0.0536 (6)	0.0006 (4)	0.0199 (5)	−0.0065 (5)
O1	0.0255 (5)	0.0245 (5)	0.0229 (5)	−0.0035 (4)	0.0071 (4)	−0.0021 (4)
O2	0.0598 (8)	0.0311 (7)	0.0356 (6)	0.0043 (6)	0.0243 (6)	0.0098 (5)
C1	0.0282 (7)	0.0225 (7)	0.0200 (6)	0.0016 (6)	0.0067 (5)	0.0014 (6)
C2	0.0252 (7)	0.0221 (7)	0.0220 (6)	0.0023 (6)	0.0071 (5)	0.0024 (6)
C3	0.0245 (7)	0.0262 (8)	0.0279 (7)	0.0001 (6)	0.0096 (5)	0.0017 (6)
C4	0.0223 (7)	0.0244 (8)	0.0327 (8)	−0.0039 (6)	0.0068 (6)	0.0001 (6)
C5	0.0270 (7)	0.0242 (7)	0.0261 (7)	−0.0004 (6)	0.0062 (5)	−0.0031 (6)
C6	0.0251 (7)	0.0230 (7)	0.0230 (6)	0.0015 (6)	0.0073 (5)	0.0009 (6)
C7	0.0226 (6)	0.0215 (7)	0.0227 (6)	−0.0016 (6)	0.0057 (5)	0.0015 (6)
C8	0.0284 (7)	0.0224 (7)	0.0203 (6)	0.0008 (6)	0.0050 (5)	0.0005 (6)
C9	0.0306 (7)	0.0230 (7)	0.0212 (6)	−0.0030 (6)	0.0042 (5)	0.0022 (6)
C10	0.0371 (8)	0.0279 (8)	0.0260 (7)	−0.0014 (7)	0.0092 (6)	0.0022 (6)
C11	0.0528 (10)	0.0258 (8)	0.0301 (8)	−0.0062 (8)	0.0090 (7)	−0.0024 (7)
C12	0.0448 (10)	0.0337 (9)	0.0352 (9)	−0.0146 (8)	0.0026 (7)	−0.0003 (7)
C13	0.0329 (8)	0.0390 (10)	0.0452 (10)	−0.0094 (8)	0.0061 (7)	0.0005 (8)
C14	0.0321 (8)	0.0291 (8)	0.0353 (8)	−0.0034 (7)	0.0071 (6)	−0.0016 (7)
C15	0.0287 (7)	0.0310 (8)	0.0266 (7)	−0.0007 (6)	0.0119 (6)	−0.0030 (6)
C16	0.0358 (8)	0.0336 (9)	0.0448 (9)	0.0079 (7)	0.0140 (7)	0.0015 (8)

### Geometric parameters (Å, º)

**Table:**

S1—O2	1.4906 (13)	C8—C9	1.460 (2)
S1—C1	1.7712 (14)	C9—C10	1.385 (2)
S1—C16	1.7892 (17)	C9—C14	1.400 (2)
Cl1—C4	1.7464 (15)	C10—C11	1.375 (2)
F1—C10	1.3554 (19)	C11—C12	1.385 (3)
O1—C7	1.3755 (17)	C11—H11	0.9500
O1—C8	1.3805 (17)	C12—C13	1.384 (3)
C1—C8	1.359 (2)	C12—H12	0.9500
C1—C2	1.446 (2)	C13—C14	1.383 (2)
C2—C7	1.3922 (19)	C13—H13	0.9500
C2—C3	1.398 (2)	C14—H14	0.9500
C3—C4	1.375 (2)	C15—H15A	0.9800
C3—H3	0.9500	C15—H15B	0.9800
C4—C5	1.402 (2)	C15—H15C	0.9800
C5—C6	1.384 (2)	C16—H16A	0.9800
C5—H5	0.9500	C16—H16B	0.9800
C6—C7	1.385 (2)	C16—H16C	0.9800
C6—C15	1.4992 (19)		
			
O2—S1—C1	105.41 (7)	C10—C9—C8	122.68 (14)
O2—S1—C16	105.47 (8)	C14—C9—C8	120.21 (14)
C1—S1—C16	98.30 (8)	F1—C10—C11	117.93 (15)
C7—O1—C8	106.28 (11)	F1—C10—C9	118.68 (14)
C8—C1—C2	107.26 (12)	C11—C10—C9	123.37 (16)
C8—C1—S1	126.15 (11)	C10—C11—C12	118.47 (16)
C2—C1—S1	125.63 (11)	C10—C11—H11	120.8
C7—C2—C3	119.11 (13)	C12—C11—H11	120.8
C7—C2—C1	104.79 (12)	C13—C12—C11	120.00 (16)
C3—C2—C1	136.09 (13)	C13—C12—H12	120.0
C4—C3—C2	116.22 (13)	C11—C12—H12	120.0
C4—C3—H3	121.9	C14—C13—C12	120.57 (17)
C2—C3—H3	121.9	C14—C13—H13	119.7
C3—C4—C5	123.95 (14)	C12—C13—H13	119.7
C3—C4—Cl1	118.62 (12)	C13—C14—C9	120.54 (16)
C5—C4—Cl1	117.43 (12)	C13—C14—H14	119.7
C6—C5—C4	120.40 (14)	C9—C14—H14	119.7
C6—C5—H5	119.8	C6—C15—H15A	109.5
C4—C5—H5	119.8	C6—C15—H15B	109.5
C5—C6—C7	115.15 (13)	H15A—C15—H15B	109.5
C5—C6—C15	123.42 (13)	C6—C15—H15C	109.5
C7—C6—C15	121.43 (13)	H15A—C15—H15C	109.5
O1—C7—C6	123.96 (13)	H15B—C15—H15C	109.5
O1—C7—C2	110.89 (12)	S1—C16—H16A	109.5
C6—C7—C2	125.15 (14)	S1—C16—H16B	109.5
C1—C8—O1	110.77 (12)	H16A—C16—H16B	109.5
C1—C8—C9	135.43 (14)	S1—C16—H16C	109.5
O1—C8—C9	113.79 (12)	H16A—C16—H16C	109.5
C10—C9—C14	117.03 (14)	H16B—C16—H16C	109.5
			
O2—S1—C1—C8	−134.26 (14)	C3—C2—C7—C6	0.8 (2)
C16—S1—C1—C8	117.09 (15)	C1—C2—C7—C6	−179.82 (14)
O2—S1—C1—C2	33.03 (14)	C2—C1—C8—O1	−0.17 (16)
C16—S1—C1—C2	−75.62 (14)	S1—C1—C8—O1	169.04 (10)
C8—C1—C2—C7	0.49 (16)	C2—C1—C8—C9	179.06 (16)
S1—C1—C2—C7	−168.79 (11)	S1—C1—C8—C9	−11.7 (3)
C8—C1—C2—C3	179.68 (16)	C7—O1—C8—C1	−0.23 (16)
S1—C1—C2—C3	10.4 (3)	C7—O1—C8—C9	−179.64 (12)
C7—C2—C3—C4	−1.2 (2)	C1—C8—C9—C10	−36.0 (3)
C1—C2—C3—C4	179.75 (16)	O1—C8—C9—C10	143.19 (14)
C2—C3—C4—C5	0.7 (2)	C1—C8—C9—C14	147.49 (18)
C2—C3—C4—Cl1	−179.56 (11)	O1—C8—C9—C14	−33.30 (19)
C3—C4—C5—C6	0.1 (2)	C14—C9—C10—F1	177.23 (13)
Cl1—C4—C5—C6	−179.61 (11)	C8—C9—C10—F1	0.6 (2)
C4—C5—C6—C7	−0.5 (2)	C14—C9—C10—C11	−1.0 (2)
C4—C5—C6—C15	179.58 (14)	C8—C9—C10—C11	−177.64 (15)
C8—O1—C7—C6	179.74 (13)	F1—C10—C11—C12	−177.97 (15)
C8—O1—C7—C2	0.56 (15)	C9—C10—C11—C12	0.3 (2)
C5—C6—C7—O1	−179.04 (13)	C10—C11—C12—C13	0.4 (3)
C15—C6—C7—O1	0.9 (2)	C11—C12—C13—C14	−0.3 (3)
C5—C6—C7—C2	0.0 (2)	C12—C13—C14—C9	−0.5 (3)
C15—C6—C7—C2	179.97 (14)	C10—C9—C14—C13	1.1 (2)
C3—C2—C7—O1	179.99 (12)	C8—C9—C14—C13	177.80 (15)
C1—C2—C7—O1	−0.65 (16)		

### Hydrogen-bond geometry (Å, º)

Cg1 is the centroid of the C1/C2/C7/O1/C8 furan ring.

**Table:**

*D*—H···*A*	*D*—H	H···*A*	*D*···*A*	*D*—H···*A*
C5—H5···O2^i^	0.95	2.44	3.2916 (19)	149
C15—H15*A*···O2^i^	0.98	2.45	3.347 (2)	152
C16—H16*B*···*Cg*1^ii^	0.98	2.92	3.641 (2)	131

Symmetry codes: (i) *x*, −*y*+1, *z*−1/2; (ii) −*x*+1/2, −*y*+3/2, −*z*+1.
